# The Reductive Leaching of Chalcopyrite by Chromium(II) Chloride for the Rapid and Complete Extraction of Copper

**DOI:** 10.1002/open.202200196

**Published:** 2023-01-04

**Authors:** Jonathan T. Vardner, Yuta Inaba, Heejung Jung, Raymond S. Farinato, D. R. Nagaraj, Scott Banta, Alan C. West

**Affiliations:** ^1^ Department of Chemical Engineering Columbia University New York NY 10027 USA; ^2^ Department of Earth and Environmental Engineering Columbia University New York NY 10027 USA

**Keywords:** chalcopyrite, copper, electrochemistry, hydrometallurgy, leaching

## Abstract

A hydrometallurgical process is developed to lower the costs of copper production and thereby sustain the use of copper throughout the global transition to renewable energy technologies. The unique feature of the hydrometallurgical process is the reductive treatment of chalcopyrite, which is in contrast to the oxidative treatment more commonly pursued in the literature. Chalcopyrite reduction by chromium(II) ion is described for the first time and superior kinetics are shown. At high concentrate loadings of 39, 78, and 117 g L^−1^, chalcopyrite reacted completely within minutes at room temperature and pressure. The XRD, SEM‐EDS, and XPS measurements indicate that chalcopyrite reacts to form copper(I) chloride (CuCl). After the reductive treatment, the mineral products are leached by iron(III) sulfate to demonstrate the complete extraction of copper. The chromium(II) ion may be regenerated by an electrolysis unit inspired by an iron chromium flow battery in a practical industrial process.

## Graphical abstract

The Cr^2+^ ion is electrochemically generated by leveraging advances in iron chromium flow battery technologies. The Cr^2+^ ion reduces chalcopyrite (CuFeS_2_) to copper(I) chloride (CuCl) for the sustainable production of copper.

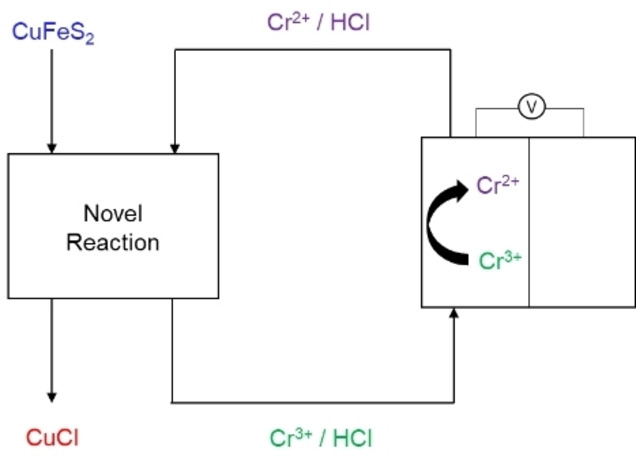

## Introduction

The increasing demand for copper coincides with declining grades of copper reserves, and consequently, a global peak in copper production is expected to arise in the coming decades.^[1]^ Alternative processing routes for chalcopyrite (CuFeS_2_), which accounts for approximately 70 % of the world's copper reserves, may extend the availability of copper throughout the 21^st^ century. The pyrometallurgical process is generally used in industry to convert CuFeS_2_ to metallic Cu despite relatively high investment and operating costs.^[2]^ The smelting step is generally considered to be environmentally deleterious due to the release of sulfur dioxide, carbon dioxide as well as the potential release of arsenic and other toxic elements. Industry and academia have sought to replace the pyrometallurgical process with a hydrometallurgical alternative for economic and environmental sustainability.^[2]^

Hydrometallurgical processes include bioleaching,[^3^, ^4^, ^5^, ^6^, ^7^, ^8^, ^9^, ^10^] high temperature and pressure leaching,[^11^, ^12^] the Galvanox process[^13^, ^14^, ^15^, ^16^] and many other variants thereof, the majority of them using ferric ion and sulfuric acid. The kinetics of CuFeS_2_ bioleaching, and leaching in general, from ores or concentrates in a sulfate (or sulfuric acid) medium are hindered by a passivating sulfur‐like or metal‐deficient layer; consequently, leaching efficiency is poor. High temperature pressure leaching overcomes the passivation, but such conditions are often uneconomical in many plants. The galvanox process is a promising alternative to enhance copper recovery at atmospheric pressure and relatively low temperature but has not seen widespread adoption by industry. It should be noted that there are other processes in various phases of development that may become promising alternatives.[^17^, ^18^]

The electrolytic conversion of CuFeS_2_ to copper may be a more promising route for its hydrometallurgical processing.^[19]^ CuFeS_2_ can be electrochemically reduced to less refractory mineral phases for copper extraction.[^20^, ^21^, ^22^, ^23^, ^24^] Equations (1) and (2) show that CuFeS_2_ can be electrochemically reduced to Cu_2_S and, subsequently, to Cu. These reactions have undergone a number of optimizations by modifying the electrolyte, separator, electrode materials, and reactor design.^[25]^

(1) $$2CuFeS_{2}+6H^{+}+2e^{-}\to {Cu}_{2}S+2{Fe}^{2+}+3H_{2}S$$

(2) $${Cu}_{2}S+{2H}^{+}+2e^{-}\to 2{Cu}^{0}+H_{2}S$$

Reactions 1 and 2 [Eqs. (1) and (2)] are in direct competition with the hydrogen evolution reaction, and therefore typically operate at Faradaic efficiencies below 40 %. These slurry reactions also present potential engineering challenges such as reactor plugging and electrode fouling.^[25]^

The chemical reduction of CuFeS_2_ may be advantageous because it obviates the hydrogen evolution reaction and circumvents engineering challenges associated with slurry electrodes. The chemical reduction of CuFeS_2_ has been tested with Fe,^[26]^ Cu,^[27]^ Al,^[28]^ and SO_2_ ^[29]^ as reductants. These reducing agents generally require high temperatures or small particle sizes, and therefore, have not been adopted by industry. In this work, Cr^2+^ was tested as a reductant for the first time and superior kinetics are demonstrated. Although the cost of chromium is high relative to copper, an electrolysis unit inspired by an iron chromium flow battery (ICFB)[^30^, ^31^, ^32^, ^33^, ^34^, ^35^] may be leveraged to efficiently regenerate the Cr^2+^ at high current densities.

## Results and Discussion

A violent reaction was observed upon adding the CuFeS_2_ concentrate to the solution of 1 m CrCl_2_ and 4 m HCl. Reaction 3 [Eqs. (3)] is postulated to be taking place and is discussed throughout this section.

(3) $$CuFeS_{2}+4H^{+}+{Cr}^{2+}\to {Cu}^{+}+{Fe}^{2+}+2H_{2}S\;+{Cr}^{3+}$$

**Figure 1:**
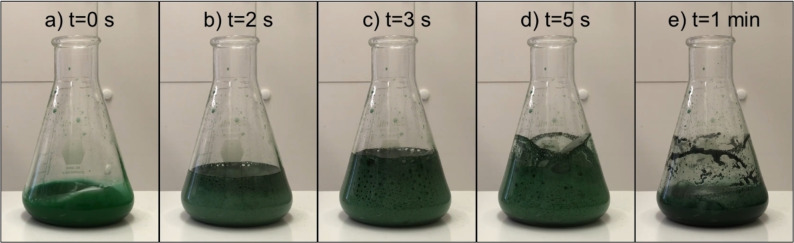
Pictures of the reaction between 1 m CrCl_2_, 4 m HCl and 78 g L^−1^ of the CuFeS_2_ concentrate at a) 0 s, b) 2 s, c) 3 s, d) 5 s, and e) 1 min.

Figure 1 shows the pictures of the reaction between 1 m CrCl_2_, 4 m HCl, and 78 g L^−1^ CuFeS_2_ concentrate after 0, 2, 3, 5, and 60 s of reaction time. The pictures show the rapid release of H_2_S gas, which was qualitatively measured with a S*ensorcon* detector. The release of gas ensued immediately upon the addition of the concentrate and concluded within a minute of reaction time. The liquid phase samples were measured with gas chromatography–mass spectroscopy (GC‐MS) to confirm the presence of dissolved H_2_S for similar experiments.

**Figure 2:**
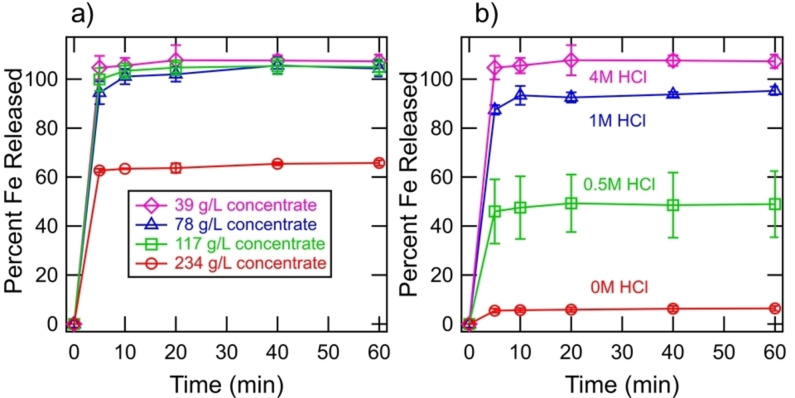
(a) Release of Fe^2+^ ions to solution during the progression of the reaction between 1 m CrCl_2_, 4 m HCl, and various loadings of CuFeS_2_ concentrate. (b) Release of Fe^2+^ ions to solution during the progression of the reaction between 1 m CrCl_2_, 39 g L^−1^ CuFeS_2_ concentrate, and various initial concentrations of HCl. Error bars show the standard deviations of replicates in triplicate.

The evolution of gaseous H_2_S coincided with the release of Fe^2+^ ions to solution, which is consistent with Reaction 3 [Eqs. (3)]. Figure 2a shows the percent of Fe^2+^ released as a function of time for a slurry comprising 1 m CrCl_2_, 4 m HCl, and CuFeS_2_ concentrate loadings of 39, 78, 117, and 234 g L^−1^. The reaction kinetics were rapid considering that approximately 100 % of Fe^2+^ was released from CuFeS_2_ within 5 min for the CuFeS_2_ concentrate loadings of 39, 78 and 117 g L^−1^. The release of Fe^2+^, however, was limited for the CuFeS_2_ concentrate loading of 234 g L^−1^ suggesting the complete utilization of Cr^2+^. Measurements of Fe^2+^ release exceeding 100 % may indicate a minor error in the estimation of composition shown in Table 1, due to both the error in XRD quantification and the sieving of the concentrate to be within 53–106 μm. The experiments were conducted while purging the headspace of the reactor with argon and similar results were observed, indicating that small amount of oxygen present in the system did not oxidize Cr^2+^ to any significant level. The release of copper ions to solution during the progression of the reaction was measured, but the quantitative results were inconsistent due to the precipitation of the ions out of solution, which is discussed below. The copper ions are thought to be released in the form of Cu^+^, rather than Cu^2+^, due to the reductive conditions of the electrolyte and presence of the chloride ion for stability. The pH of the solutions after the reduction experiments were below zero, ensuring that these reactions were not pH limited. The Cu^2+^ ion is not thought to be present due to the reductive conditions in the electrolyte.

**Table 1:** Mineralogy of concentrate supplied by Freeport‐McMoRan.

Mineral	Chemical Formula	Percent
Chalcopyrite	CuFeS_2_	78.3
Pyrite	FeS_2_	12.9
K‐feldspar	KAlSi_3_O_8_	2.9
Plagioclase	NaAlSi_3_O_8_	2.9
Quartz	SiO_2_	2.2
Molybdenite	MoS_2_	0.85

Figure 2b shows the percent of Fe^2+^ released as a function of time for slurries comprising 1 m CrCl_2_, 39 g L^−1^ of CuFeS_2_ concentrate, and initial HCl concentrations of 0 m, 0.5 m, 1 m, and 4 m. The pH of the solution after the reduction step was approximately 2.5 for the slurries with initial HCl concentrations of 0 m, 0.5 m, 1 m, indicating that these reactions were pH limited. The pH of the solution after the reduction step may be leveraged to facilitate a separation between Fe^2+^ and Cr^3+^, which may be desirable prior to the reduction of Cr^3+^ to Cr^2+^ by an electrolysis unit. These results suggest that the proton has a greater stoichiometric number than CuFeS_2_, which is consistent with Reaction 3 [Eqs. (3)]. The experiments conducted with initial HCl concentrations of 2 m and 3 m were found not to be pH limited.

**Figure 3:**
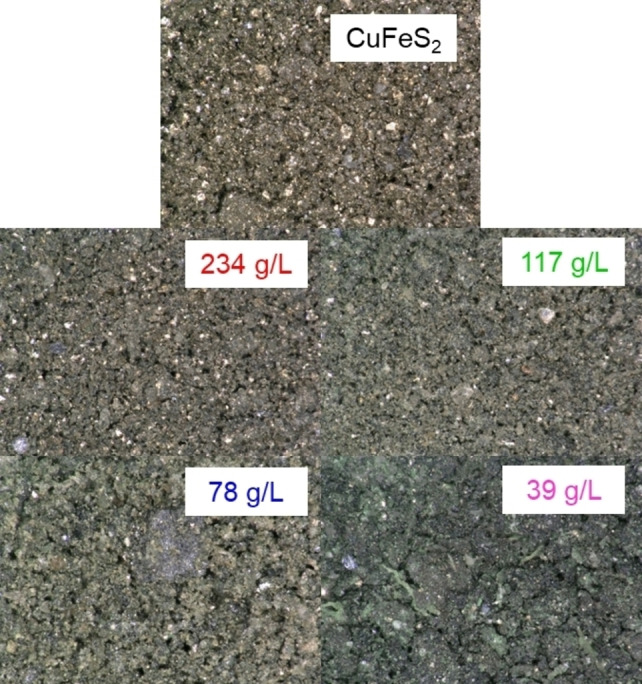
Optical microscopy images of the mineral products after reaction between various chalcopyrite concentrate loadings, 1 m CrCl_2_ and 4 m HCl for 60 min.

Figure 3 shows images of the mineral products after 60 min of reduction with the Cr^2+^ ion obtained with a *Keyence VHX‐5000* microscope. The results indicate that the mineral product is affected by the CuFeS_2_ concentrate loading. The 39 g L^−1^ CuFeS_2_ loading yielded a green product, which is consistent with the appearance of CuCl as well as other potential Cu−Cl complexes. The various mineral products were characterized and shown to yield different amounts of copper recovery, which is discussed below. The mineral products post reaction with various HCl concentrations yielded the same trend in appearance.

**Figure 4:**
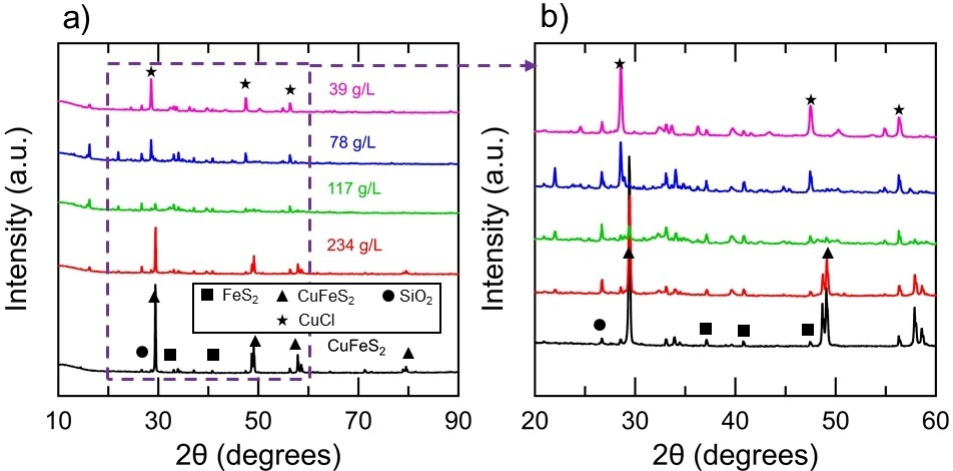
XRD results for the mineral products after reaction between various chalcopyrite concentrate loadings, 1 m CrCl_2_ and 4 m HCl for 60 min. (b) Close‐up of region used to identify mineral products.

**Figure 5:**
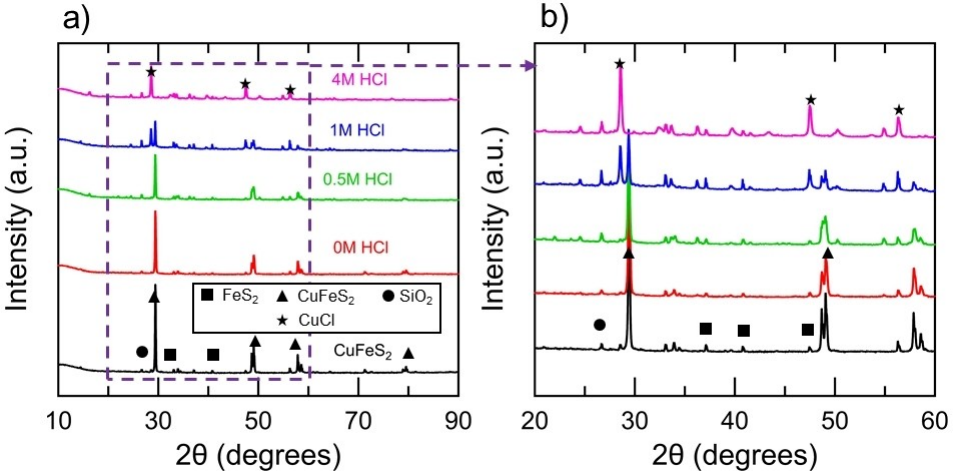
(a) XRD results for the mineral products after reaction between 39 g L^−1^ of the chalcopyrite concentrate with 1 m CrCl_2_ and various initial concentrations of HCl for 60 min. (b) Close‐up of region used to identify mineral products.

Figure 4 shows the XRD spectra for the various chalcopyrite concentrate loadings subsequent to reaction with the Cr^2+^ ion, and Figure 5 shows the XRD spectra for the mineral samples subsequent to reaction with the Cr^2+^ ion and various initial HCl concentrations. The predominant peaks of the unreacted CuFeS_2_ concentrate were consistent with CuFeS_2_, FeS_2_, and SiO_2_, as shown in Table 1. The relative intensity of the peaks associated with CuFeS_2_ diminished for the reacted mineral products, consistent with the Fe^2+^ release measured by AAS. The peaks associated with the reaction products emerged for the mineral products with high conversion of CuFeS_2_. The predominant mineral product was determined to be copper chloride (CuCl) from the spectra. Secondary products, such as Cu_2_(OH)_3_Cl, were consistent with the spectra. Reaction 4 [Eqs. (4)] shows the precipitation of CuCl out of solution, which is the primary product formed. Reaction 4 is shown for simplicity whereas the chemistry taking place is more complicated and a variety of Cu−Cl complexes precipitate. The precipitation of CuCl out of the solution containing 4 m HCl was unexpected considering that the molar ratio of Cl/Cu was 36 in the system. However, the molar ratio of Cl/Cr was 6, and therefore, complexes formed between Cl^−^ and Cr^3+^ may lower the number of Cl^−^ ions available to stabilize Cu^+^. The concentration of Cu^+^ in solution after 60 min of reduction was approximately 0.07 m, which is close to the solubility limit of 0.233 m reported at 2 m HCl in the literature.^[36]^ It is estimated that 40 % of copper in the system remained in the bulk solution as Cu^+^ and 60 % precipitated out of solution for the experiments conducted with a concentrate loading of 39 g L^−1^ and an acid concentration of 4 m HCl.

(4) $${Cu}^{+}+{Cl}^{-}\to CuCl$$

The XRD data, in conjunction with the AAS data, indicate that the FeS_2_ and silicates were inert during the reductive treatment. Experiments were conducted between 39 g L^−1^ CuFeS_2_ concentrate, 1 m CrCl_2_, 4 m HCl and initial ferrous chloride (FeCl_2_) concentrations of 0, 0.5 m, 1 m, and 2 m. It was determined that the reduction process can tolerate initial FeCl_2_ concentrations of 1 m and below. The Fe^2+^ precipitated out of solution for the experiment conducted with an initial FeCl_2_ concentration of 2 m.

**Figure 6:**
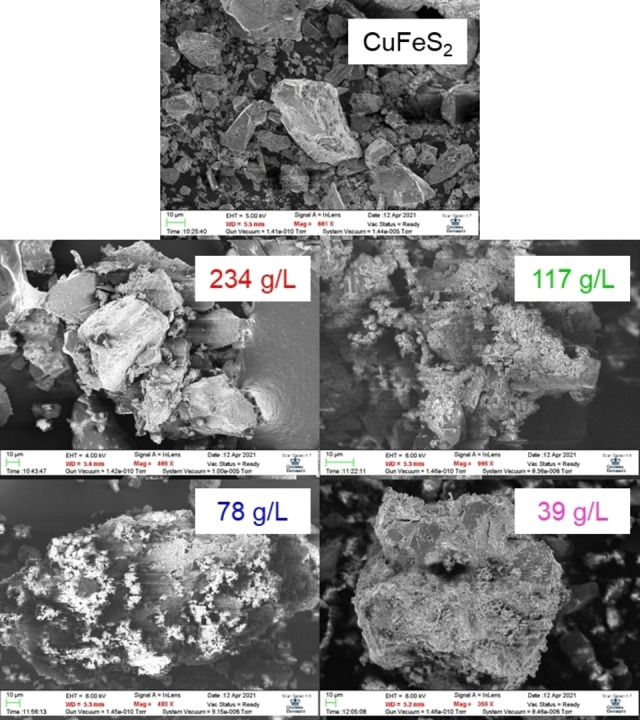
SEM images of mineral products after reaction with 1 m CrCl_2_ and 4 m HCl for 60 min.

**Figure 7:**
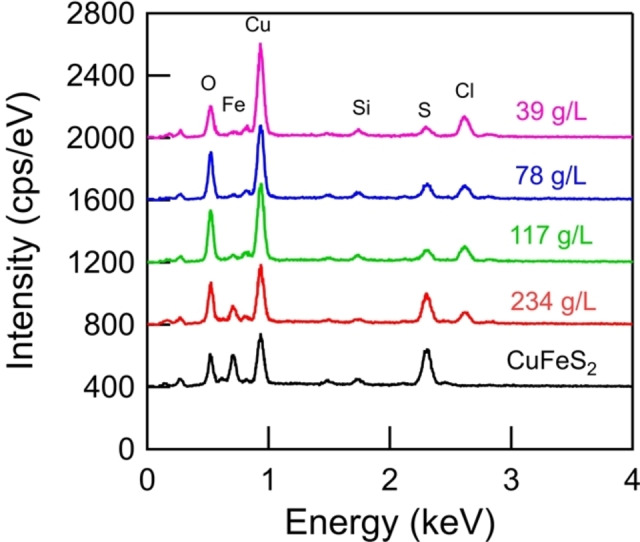
EDS results for the mineral products after reaction with 1 m CrCl_2_ and 4 m HCl for 60 min.

Figure 6 shows SEM results for the mineral products after reaction with 1 m CrCl_2_ and 4 m HCl for 60 min. The mineral products develop some mossy features, which may be related to the growth of CuCl. Figure 7 shows EDS results for the mineral samples post reduction with the Cr^2+^ ion. The unreacted CuFeS_2_ concentrate samples show peaks corresponding to Cu, Fe, S, Si, and O. The reacted samples show the diminishment in the Fe and S peaks, which is consistent with the release of Fe^2+^ to solution and the release of H_2_S as a gas. The minor S peak present in the 39 g L^−1^ sample may be related to the presence of unreacted FeS_2_ in the mineral products. The reacted samples also show the emergence of the Cl peak, which is consistent with the formation of CuCl. The Cu peak elongates for the reacted samples due to the increasing mass fraction of Cu within the samples. No peak corresponding to Cr was observed in the spectra, indicating that the presence of Cr within the samples is minor. The samples were digested in aqua regia and the mass fraction of Cr within the samples was estimated to be 1–3 %. The presence of chromium is thought to be an artifact of the procedure used to filter and dry the mineral products.

**Figure 8:**
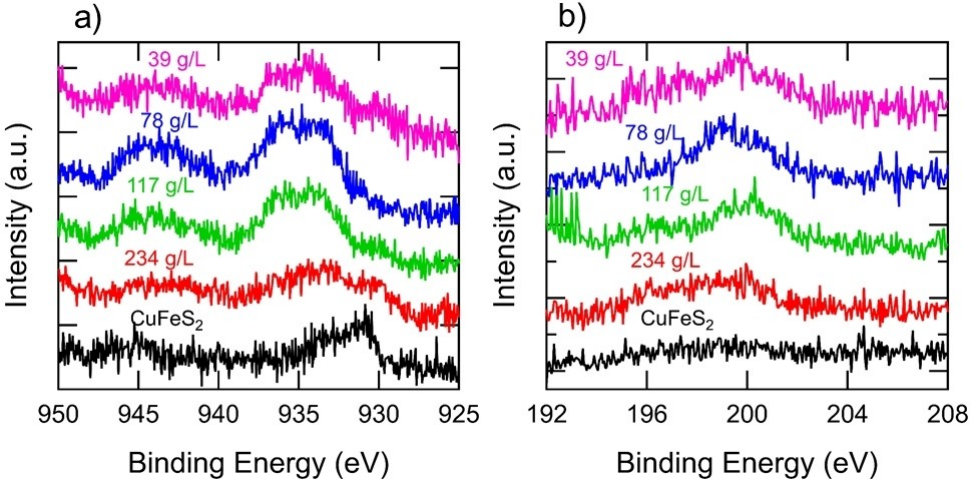
XPS results for mineral products after reaction with 1 m CrCl_2_ and 4 m HCl for 60 min for (a) Cu and (b) Cl.

Figure 8 shows the XPS spectra of Cu and Cl for the mineral samples post reduction with the Cr^2+^ ion. The Cr element was not observed on the mineral products, which further indicates that the samples were not comprised of chromium. Similarly, Fe and S were not observed on the surface of the mineral reaction products, which is consistent with the release of Fe^2+^ and H_2_S from the surface of the particles into the solution phase. The XPS data indicate the absence of a sulfur passivation layer, which may account for the rapid kinetics of the reduction reaction. The various copper peaks indicate the presence of several copper‐containing products leading to convoluted spectra. For instance, the peaks at the binding energies of 944 and 935 eV are assigned to Cu_2_(OH)_3_Cl and CuCl, respectively. The Cu scans also show an observable shift in binding energy from the CuFeS_2_ concentrate standard. The emergence of a Cl peak for the reacted samples is consistent with the formation of Cu−Cl complexes.

**Figure 9:**
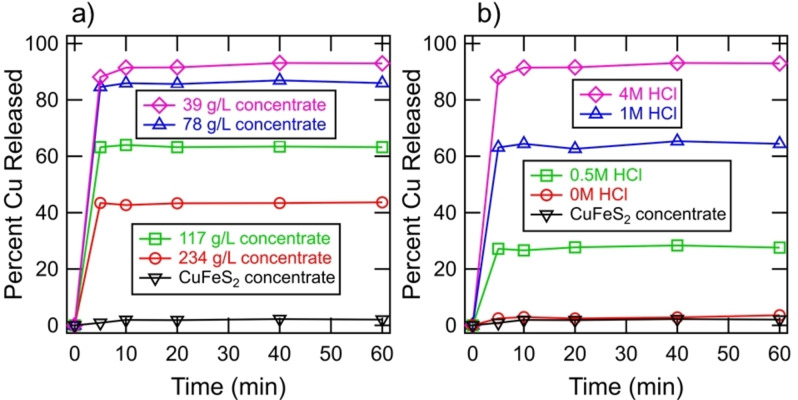
(a) Extraction of Cu^2+^ from mineral products by 0.5 m Fe_2_(SO_4_)_3_ subsequent to the reaction between 1 m CrCl_2_, 4 m HCl, and various loadings of CuFeS_2_ concentrate. (b) Extraction of Cu^2+^ from mineral products by 0.5 m Fe_2_(SO_4_)_3_ subsequent to reaction between 1 m CrCl_2_, 39 g L^−1^ CuFeS_2_ concentrate, and various initial concentrations of HCl.

Figure 9 shows the extraction of Cu^2+^ from the mineral products by 0.5 m Fe_2_(SO_4_)_3_. Reaction 5 [Eqs. (5)] shows the leaching reaction of CuCl by the Fe^3+^ oxidant, which goes to completion within minutes.

$$CuCl+{Fe}^{3+}\to {Cu}^{2+}+{Cl}^{-}+{Fe}^{2+}$$

The results show that virtually all of the Cu^2+^ can be extracted from the 39 g L^−1^ mineral product. The aqueous solution may subsequently undergo solvent extraction and electrowinning for the production of metallic copper. In experiments, not shown, the 39 g L^−1^ sample was solubilized to the same extent in 1 m H_2_SO_4_, and therefore, the ferric ion may not be required for the extraction of copper. The incomplete copper extraction for higher pulp densities is partly related to the incomplete conversion of CuFeS_2_ shown in Figure 2. Also, potential intermediates formed, such as Cu_2_(OH)_3_Cl, may be refractory for copper leaching and undesirable. It is shown that virtually no Cu^2+^ is extracted from the CuFeS_2_ concentrate, and therefore, the reductive treatment directly leads to the extraction of copper.

## Conclusions

Chalcopyrite concentrate was reduced by CrCl_2_ in acid solution for the first time and superior kinetics were shown at room temperature and ambient pressure. AAS was used to measure the complete release of Fe^2+^ from CuFeS_2_ within minutes during its reduction. XRD and SEM‐EDS were used to characterize the predominant mineral product to be CuCl. The measurements also indicate that pyrite and silicates were inert during the reductive treatment. XPS was used to measure the surface of the mineral products and the results suggest that the rapid kinetics may be related to the lack of a passivation layer during the reduction step. The mineral products were leached by the ferric ion to demonstrate complete copper recovery.

## Experimental Section

### Reduction of CuFeS_2_

Chalcopyrite mineral concentrate was kindly provided by *Freeport‐McMoRan*. It was analyzed by the supplier with energy dispersion X‐ray diffraction to have the following composition as shown in Table 1.

The CuFeS_2_ concentrate was sieved (−140+270 mesh) to confine the particle size to be within 53–106 μm. An amount of 50 g of the concentrate was subsequently rinsed with 1 L of DI water and 1 L of 1 m H_2_SO_4_ to remove any soluble iron and copper ions generated during natural concentrate oxidation occurring in transport and storage. CuFeS_2_ concentrate pulp densities of 39, 78, 117, or 234 g L^−1^ were added to a 250 mL Erlenmeyer flask containing 25 mL of a solution comprising of 1 m CrCl_2_ and 4 m HCl. For other experiments, a CuFeS_2_ concentrate pulp density of 39 g L^−1^ was added to a solution comprising 1 m CrCl_2_ and various HCl concentrations. Thirdly, for other experiments, a CuFeS_2_ concentrate pulp density of 39 g L^−1^ was added to a solution comprising of 1 m CrCl_2_, 4 m HCl, and various concentrations of FeCl_2_. It was imperative for the reaction to be conducted in a fume hood due to the rapid release of H_2_S gas, as shown in Figure 1. Liquid phase 100 μL samples were taken at time points of 0, 5, 10, 20, 40, and 60 min, which were subsequently diluted for the measurements of Fe^2+^ and Cu^+^ contents. After the reduction, the mineral particles were filtered from solution and allowed to air dry prior to characterization.

### Atomic Absorption Spectroscopy (AAS)

An *iCE 3300* AAS was used to measure the release of Fe^2+^ and Cu^+^ ions into solution from CuFeS_2_ during its reduction. The characteristic wavelengths for the iron and copper measurements were 248.3 nm and 324.8 nm, respectively. Standards ranging from 0–4 ppm were measured immediately before the samples to construct linear (R^2^>0.995) calibration curves.

### X‐ray Diffraction (XRD)

A PANalytical XPert3 Powder XRD was used to measure the bulk mineral phase of the reaction products. The XRD was operated with filtered Empyrean Cu *K*a radiation (k=0.15418 nm), a tube voltage of 45 kV, and a current of 40 mA. The mineral products were placed on a silicon crystal zero‐diffraction plate (MTI Corporation) and were adhered in place with Apiezon grease. The samples were scanned continuously in the range of 10–100*°* with a step size of 0.0065*°* on a spinning plate with a revolution time of 2.0 s. A PIXcel1D detector was used to record the peak intensity for the subsequent analysis of the mineral composition.

### X‐ray Photoelectron Spectroscopy (XPS)

A *PHI 5500* XPS equipped with an Al X‐ray source was used to measure the elemental composition of the reaction product surfaces. The base pressure of the chamber was approximately 1×10^−8^ torr. Samples were supported on carbon tape.

### Scanning Electron Microscopy – Energy Dispersion X‐ray Spectroscopy (SEM‐EDS)

A *Zeiss Sigma VP* SEM was used to capture images of the mineral products after reaction. The SEM‐EDS analysis was operated at an accelerating potential of 6 *k*V and base pressure of approximately 1×10^−5^ torr. Samples were supported on carbon tape and were coated with gold using a *Cressington 108 Auto Sputter Coater*. The sputtering was conducted under argon gas flow with 0.1 mbar of pressure for 20 s. A *Bruker XFlash Detector* was used for EDS analysis to analyze elemental composition.

### Subsequent Leaching of Mineral Products

A sample of the mineral products was digested in aqua regia for complete copper extraction, and an equivalent sample of the mineral products was leached in a solution comprising 0.5 m Fe_2_(SO_4_)_3_ in 1 m H_2_SO_4_. The percent of copper released was determined by the ratio of copper extracted by the two leachants.

## Conflict of interest

The authors declare no conflict of interest.

1

## Data Availability

Research data are not shared.
